# Exploring the Potential Imaging Biomarkers for Parkinson’s Disease Using Machine Learning Approach

**DOI:** 10.3390/bioengineering12010011

**Published:** 2024-12-27

**Authors:** Illia Mushta, Sulev Koks, Anton Popov, Oleksandr Lysenko

**Affiliations:** 1 Department of Electronic Computational Equipment Design, National Technical University of Ukraine “Igor Sikorsky Kyiv Polytechnic Institute”, 03056 Kyiv, Ukraine; o.m.lysenko@kpi.ua; 2 Perron Institute for Neurological and Translational Science, Murdoch University, Nedlands, WA 6009, Australia; sulev.koks@perron.uwa.edu.au; 3 Department of Electronic Engineering, National Technical University of Ukraine “Igor Sikorsky Kyiv Polytechnic Institute”, 03056 Kyiv, Ukraine; popov-ee@lll.kpi.ua; 4 Faculty of Applied Sciences, Ukrainian Catholic University, 79026 Lviv, Ukraine

**Keywords:** Parkinson’s disease, DATSCAN, machine learning, basal ganglia, classification, AdaBoost

## Abstract

Parkinson’s disease (PD) is a neurodegenerative disorder characterized by motor and neuropsychiatric symptoms resulting from the loss of dopamine-producing neurons in the substantia nigra pars compacta (SNc). Dopamine transporter scan (DATSCAN), based on single-photon emission computed tomography (SPECT), is commonly used to evaluate the loss of dopaminergic neurons in the striatum. This study aims to identify a biomarker from DATSCAN images and develop a machine learning (ML) algorithm for PD diagnosis. Using 13 DATSCAN-derived parameters and patient handedness from 1309 individuals in the Parkinson’s Progression Markers Initiative (PPMI) database, we trained an AdaBoost classifier, achieving an accuracy of 98.88% and an area under the receiver operating characteristic (ROC) curve of 99.81%. To ensure interpretability, we applied the local interpretable model-agnostic explainer (LIME), identifying contralateral putamen SBR as the most predictive feature for distinguishing PD from healthy controls. By focusing on a single biomarker, our approach simplifies PD diagnosis, integrates seamlessly into clinical workflows, and provides interpretable, actionable insights. Although DATSCAN has limitations in detecting early-stage PD, our study demonstrates the potential of ML to enhance diagnostic precision, contributing to improved clinical decision-making and patient outcomes.

## 1. Introduction

Parkinson’s disease (PD) is a chronic, progressive, long-term, neurodegenerative disorder of the central nervous system with no currently effective cure. It is characterized by a growing number of cases worldwide, with prevalence increasing in part due to higher life expectancy. Studies estimate that global cases of PD rose from 2.5 million in 1996 to over 6.1 million in 2016 [1]. Approximately one in every hundred individuals over the age of 65 is affected by PD [2]. While the disease predominantly affects older adults, it does not spare younger populations. Age is the primary risk factor for developing PD, with men being more prone to the condition than women, at an estimated prevalence ratio of 3:2 [3]. Despite its prevalence, PD remains undetected in 10–20% of cases and is misdiagnosed in about 25% of instances [2]. This is due to the variable progression of symptoms among patients and the difficulty of distinguishing PD from other conditions in its early stages. PD impacts both motor and non-motor functions. Symptoms typically develop gradually and intensify over time. As the disease advances, non-motor symptoms become more prevalent [4]. Common motor symptoms include hand tremors, limb rigidity, bradykinesia (slowness of movement), gait and balance difficulties, postural instability, and stiffness (hypertonia). Non-motor symptoms may involve dementia, olfactory loss, falls, changes in voice, and neuropsychiatric issues, such as sleep disturbances, psychosis, mood fluctuations, reduced motivation, depression, apathy, social anxiety disorder, behavioral changes, and slowed cognitive processing speed [5]. As the motor symptoms of PD become more manageable, the non-motor symptoms emerge as the primary issue in PD patient care. It is also observed that non-motor symptoms of PD appear before the motor symptoms, complicating early diagnosis [6]. Additionally, none of these symptoms are definitive. This is why there is a trend in PD detection to combine multiple patient symptoms, features, and medical measurements.

The authors of manuscript [7] employed various machine learning (ML) algorithms, including multilayer perceptron [8], Bayesian network [9], random forest [10], boosted logistic regression [11,12], utilizing non-motor features and biomarkers such as the University of Pennsylvania Smell Identification Test and SPECT data. Boosted logistic regression achieved the best results, with 97.16% accuracy and a 98.9% area under receiver operating characteristic curve (ROC). Similarly, research [13] primarily focused on voice data, utilizing models such as logistic regression and random forest while addressing dataset imbalance and model performance metrics. The random forest model achieved 98.3% accuracy and a perfect 100% ROC. In manuscript [14] support vector machines [15] applied to voice signals achieved 80% accuracy and ROC. Features like frequency and amplitude were extracted from 40 participants (20 healthy, 20 PD). Study utilized techniques like the synthetic minority oversampling technique (SMOTE) [16], feature selection, and grid search cross-validation technique (GridSearchCV) [17]. The multilayer perceptron model reached 98% accuracy, 98% recall, and 100% precision, using data from 31 participants (twenty-three PD, eight healthy). The approach of using multiple ML models and feature combinations was extensively applied in study [18]. The authors performed a huge data preprocessing step, presenting the histogram and Pearson correlation coefficients between the considered features. The proposed ensemble network achieved the highest accuracy over all considered ML models, reaching 96.68% accuracy and 98.9% area under the ROC curve. The proposed boosting methods, BOOST_GAM, BOOST_GLM, and BOOST_TREE, closely followed deep learning (DL) techniques, each achieving accuracy above 96.2%. The study emphasized the high potential of the presented DL mode, particularly in handling increasingly large and complex datasets over time. A significant finding of this work was demonstrating that individuals with lower values of PUTMEN_L, PUTMEN_R, and UPSIT scores have a higher tendency to develop PD. In the manuscript by [19], the authors introduced a DL-based model to detect PD using gait and speech impairment motor symptoms. The proposed framework comprises two modules: a VGFR spectrogram detector, which leverages distorted walking patterns, and a voice impairment classifier, which focuses on speech impairments in PD patients. Accuracy comparisons were performed across several ML models, including support vector machines (SVM), XGBoost, and multilayer perceptrons (MLP). The VGFR spectrogram detector achieved the highest accuracy of 88.17% using DL, outperforming SVM (86.12%), XGBoost (78.66%), and MLP (87.79%). Similarly, the voice impairment classifier obtained 89.15% accuracy with the DL model, surpassing SVM (81.16%), XGBoost (77%), and MLP (85.60%). The authors of the manuscript [20] proposed classifying dopamine transporter scan (DATSCAN) images using the Visual Geometry Group 16 convolutional neural network (VGG16 CNN) [21] with transfer learning, achieving 95.2% accuracy. The local interpretable model-agnostic explainer (LIME) demonstrated that healthy regions influenced the classification of non-PD patients. In the manuscript [22], eye-tracking data from saccade experiments were used to classify PD through advanced DL methods. Using a dataset of 84 participants (54 PD patients, 30 healthy controls), the study utilized raw 1.5 s fixation intervals instead of hand-crafted features. Two models, InceptionTime and ROCKET, achieved 78% and 88% accuracy, respectively. Finally, this recent work [23] provides a comprehensive review of the performance of various ML methods for PD recognition, offering a high-level overview of the current state of ML applications in the PD domain. It underscores the high performance of different ML algorithms in PD diagnosis.

While ML techniques have been increasingly applied to PD diagnosis, many studies focus on either non-motor features or voice data [24] or combine various clinical biomarkers with neuroimaging, with limited emphasis on neuroimaging data alone [25]. There is a growing need to explore how specific neuroimaging biomarkers, such as the striatal binding ratio (SBR) parameters from DATSCAN, can enhance PD detection. Previous research has highlighted the effectiveness of combining different data sources [26], but few studies have concentrated solely on the potential of DATSCAN imaging for feature extraction and machine learning-based classification [27,28].

To address this gap, our research aims to enhance the diagnostic process for PD by identifying the most effective ML classifier and a single biomarker from DATSCAN imaging data. While many existing studies combine various biomarkers and ML techniques for PD diagnosis, this research focuses exclusively on SBR parameters from DATSCAN to simplify the diagnostic process and ensure high accuracy. The methodology includes applying several ML classifiers that have demonstrated effectiveness in PD classification [23]: support vector classifier (SVC), decision tree classifier, k-nearest neighbors classifier (KNeighborsClassifier), logistic regression classifier, stochastic gradient descent classifier (SGDClassifier), AdaBoost classifier, and random forest classifier. A GridSearchCV was employed to evaluate and compare the performance of these models, ultimately identifying the best-performing classifier and the most predictive SBR biomarker for accurate PD diagnosis. This approach seeks to streamline the diagnostic process by reducing the number of necessary parameters while maintaining a high level of diagnostic precision.

The contributions of this paper are as follows:

1. Focus on neuroimaging data: concentrates exclusively on striatal binding ratio (SBR) parameters from DATSCAN, addressing a gap in PD diagnosis research by limiting the scope to neuroimaging biomarkers;
2. Identification of a single biomarker: demonstrates that a single SBR parameter can achieve high diagnostic accuracy, reducing the need for complex feature combinations and simplifying the diagnostic process;
3. Evaluation of ML classifiers: comprehensively evaluates and compares multiple ML classifiers, identifying AdaBoost as the best-performing model for this specific dataset;
4. Streamlining PD diagnosis: provides a minimally invasive, highly accurate diagnostic method by reducing the number of required biomarkers and focusing on interpretability and practical application;
5. Comparison to existing methods: highlights the advantages of the proposed approach over previous studies that used diverse data sources, including non-motor features and voice data, or combined multiple biomarkers and machine learning techniques;
6. Gender-specific analysis: separates the dataset by gender to independently evaluate classification performance for male and female participants, confirming that con_putamen remains a reliable biomarker across genders and showing no significant performance differences between the two groups.

By simplifying the diagnostic approach to rely on one highly predictive biomarker, we aim to facilitate earlier and more accurate PD diagnosis, offering a practical and minimally invasive method that compares favorably to existing PD classification approaches.

The structure of this paper is organized as follows: Section 2 discusses the alterations in brain structures caused by PD and the diagnostic significance of DATSCAN imaging. Section 3 outlines the dataset and the classification methodology used. The results are presented in Section 4, while Section 5 provides a detailed discussion. Finally, Section 6 concludes the paper.

## 2. Background

**Figure 1:**
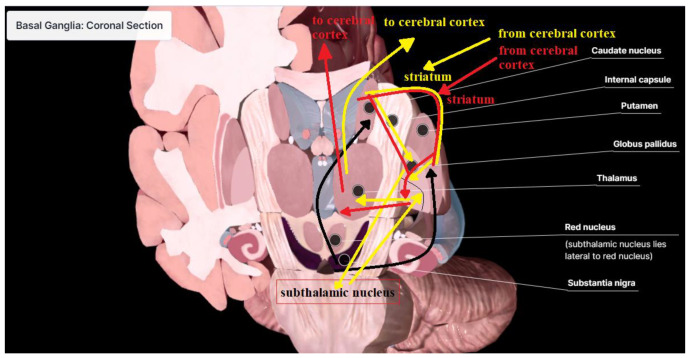
Direct pathway (red), indirect pathway (yellow), and nigrostriatal pathway (black).

The primary brain structure implicated in PD is the basal ganglia. More generally, basal ganglia is a group of interconnected subcortical nuclei, spanning the telencephalon, diencephalon, and midbrain [29]. The basal ganglia and related nuclei can be divided into three distinct parts—input nuclei, output nuclei, and intrinsic nuclei [30]. Input nuclei receive information from cortical, thalamic, and nigral structures. They consist of the caudate nucleus (CN), the putamen (Put), and the nucleus accumbens (Acb). The output nuclei send basal ganglia information to several regions, with the most important being the ventral tier (ventral-anterior and ventral-lateral; VA/VL) and the mediodorsal (MD) thalamic nuclei [29]. They consist of globus pallidus internal (GPi) and substantia nigra pars reticulata (SNr) structures. The intrinsic nuclei are the globus pallidus external (GPe), subthalamic nucleus (STN), and substantia nigra pars compacta (SNc) structures that mediate information flow between input and output nuclei. To describe the connections between CN, Put, Acb, GPi, GPe, SNc, SNr, STN, and other structures, multiple basal ganglia models are created. The central components of each basal ganglia model that describe its functionality are the direct and indirect pathways shown in Figure 1 [31]. The direct pathway is formed by medium spiny neurons (MSNs) projections from the Put to GPi/SNr. MSNs constitute 90% of striatal neurons. They possess dopamine D1-type receptors, express peptides like substance P and dynorphin, and exert a direct inhibitory influence on GPi/SNr neurons [32]. An indirect pathway is formed by MSNs connecting the Put with the GPi/SNr via synaptic connections in the GPe and STN. They harbor D2 receptors along with the peptide enkephalin (ENK) [33]. The projections from the Put to the GPe and from the GPe to the STN are gamma-aminobutyric acid (GABA) releasing and exert inhibitory effects [33]. Dopamine (DA), a brain hormone, is synthesized by dopaminergic neurons in the nigrostrial system originating from the SNc, which have axon projections in the striatum [34]. As a neurotransmitter, DA is released from the presynaptic membrane into the synaptic cleft, where it binds to and activates DA receptors on the postsynaptic membrane. DA has a dual effect on MSNs, inhibiting neurons in the striatum that contain D2 receptors while simultaneously exciting neurons in the striatum that express D1 receptors via L-type calcium channels [30]. DA plays an important role in a precise selection mechanism to filter incoming and outgoing signals to perform precise movements. Progressive degeneration of dopaminergic neurons decreases DA levels in the SN and striatum, leading to the onset of clinical symptoms of PD [34]. Neurons that remain viable frequently exhibit intracellular accumulations of misfolded proteins, with alpha-synuclein being the most notable among them. These aggregates form circular-shaped structures called Lewy bodies, which are recognized as the histopathological signature of PD [30]. Stimulation of neurons within the indirect pathway results in the inhibition of the GPe, leading to disinhibition of the STN and excitation of the GPi/SNr [32]. Consequently, the overall activity of the basal ganglia output is shaped by the contrasting effects of inhibitory inputs from the direct pathway and excitatory inputs from the indirect pathway. Thus the direct and indirect pathways exhibit opposing influences on the output function of the basal ganglia. In general, the basal ganglia tend to favor the inhibition of movements facilitated by the cortex due to heightened activation of the STN–GPi network and diminished excitability in the direct cortico-putaminal-GPi projection [30].

To notice changes in described brain structures affected by PD, there are many imaging diagnosis methods available, including positron emission tomography (PET), magnetic resonance imaging (MRI), functional magnetic resonance imaging (fMRI), and others. However, SPECT is a technique that is most frequently used for the early diagnosis of PD [35]. The DATSCAN variant of SPECT, which uses 123I-ioflupane (also known as 123I-FP-CIT), is particularly effective for investigating the loss of dopaminergic neurons in the striatum. The principle of the DATSCAN involves the radiopharmaceutical ioflupane (123I), which binds to dopamine transporters (DAT). In PD patients, the absence of dopaminergic neurons leads to a noticeable reduction in the Put and CN areas, which is detectable by DATSCAN [36]. This is why in this research we are mostly focusing on 13 SBR-based parameters taken from a DATSCAN image.

## 3. Materials and Methods

### 3.1. Data

The Parkinson’s Progression Markers Initiative (PPMI) database [37] is used for this research. The PPMI is a comprehensive observational study designed to evaluate specific cohorts through advanced imaging, biological samples, and thorough clinical and behavioral evaluations to identify biomarkers that indicate the progression of PD. Participants were carefully selected to represent a diverse group, including newly diagnosed, untreated PD patients, healthy controls, and individuals at risk of developing the disease. Eligibility criteria required participants to be over the age of 30, with a clinical diagnosis of PD based on the UK Parkinson’s Disease Society Brain Bank criteria for PD patients, and for healthy controls, no neurological conditions that could affect the study’s outcome. The PPMI contains three main data collections—PPMI clinical, PPMI remote, and PPMI online [38]. PPMI clinical collection contains data gathered through clinical assessments, non-motor assessments, movement disorder society unified PD rating scale (MDS-UPDRS) questionnaires, biospecimen analysis, MRI and DATSCAN analysis, medical history, and genetic test results. PPMI clinical data are targeted to cover 4000 participants. The PPMI remote collection contains remote assessment data gathered mostly for participants with a pre-diagnostic phase of PD. The PPMI remote gathers data mainly related to olfactory symptoms aiming to utilize these data for the early detection of PD by identifying potential early signs of the disease. PPMI remote data are targeted to cover 40,000 participants. Participants’ self-reported data amounts are contained in the PPMI online data collection. The PPMI clinical and PPMI online datasets share certain data, encompassing the first two sections of MDS-UPDRS questionnaires. Additionally, the PPMI online collection includes participants with a confirmed diagnosis of PD as well as those without confirmed diagnoses. PPMI online data are targeted to cover 4000 participants.

PPMI clinical participants are divided into five cohorts [38]:

1. Parkinson’s Disease—people who have a formal diagnosis of PD;
2. Prodromal—people who are at risk of developing PD based on clinical features, genetic variants, or other biomarkers but have not been formally diagnosed;
3. Healthy controls—people with no neurologic disorder and no first-degree relative with PD;
4. SWEDD—people having scans without dopaminergic deficit;
5. Early imaging—a cohort of participants with a confirmed diagnosis of PD, who were untreated and underwent additional tests including DATSCAN and AV-133 imaging.

This research uses a curated dataset provided by PPMI that consists of PPMI clinical data comprising 2347 participants with up to 12 annual study visits. For the PD detection task, we divided participants into two main groups: healthy individuals (healthy controls) and those diagnosed with PD. Excluding the SWEDD, early imaging and prodromal groups, and focusing on participants’ study visits while removing entries with missing data, the final dataset consists of 2422 PD observations and 257 healthy control observations.

**Figure 2:**
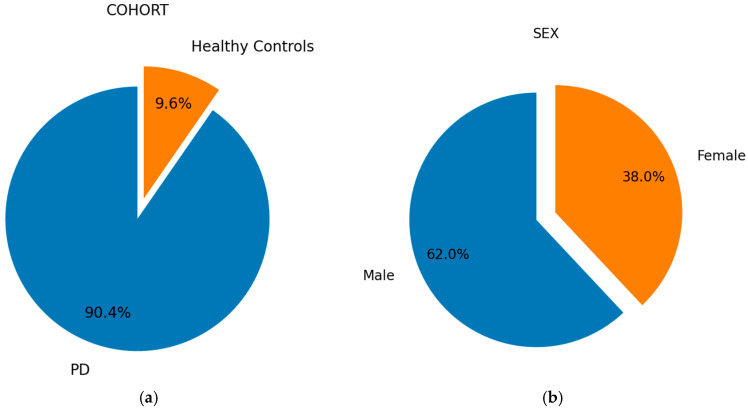

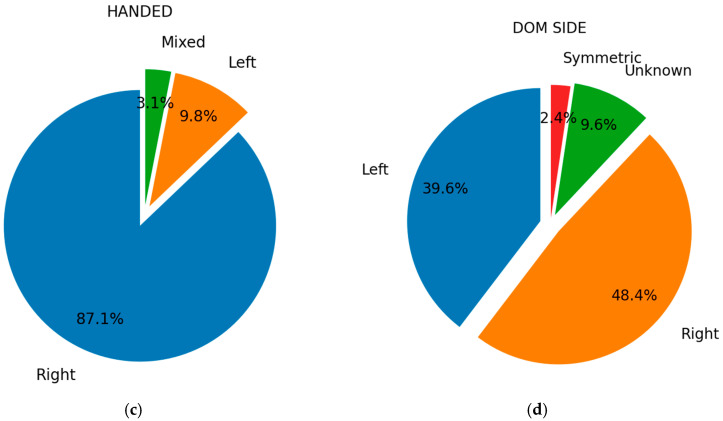
The distribution of key demographic and clinical characteristics of the participants: (**a**) cohort at enrollment; (**b**) sex at birth; (**c**) handedness; (**d**) side most affected at PD symptom onset.

The COHORT, SEX, HANDED, AND DOM SIDE pie charts are presented in Figure 2 to provide a comprehensive overview of the dataset and visualize the distribution of key demographic and clinical characteristics of the participants.

The pie charts reveal that the dataset is predominantly composed of individuals diagnosed with PD, accounting for 90.4% of participants, while only 9.6% are healthy controls. This notable class imbalance must be addressed to ensure an accurate diagnosis. Additionally, the gender distribution shows a considerable disparity, necessitating an analysis of its impact on the model’s predictive performance. Handedness, which can influence symptom presentation in motor diseases like PD, should also be carefully considered when interpreting the classification results.

**Figure 3:**
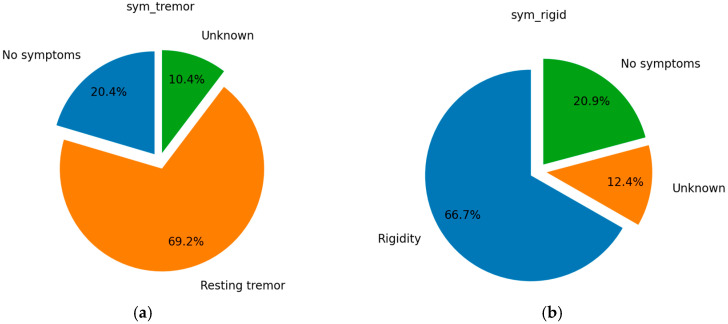

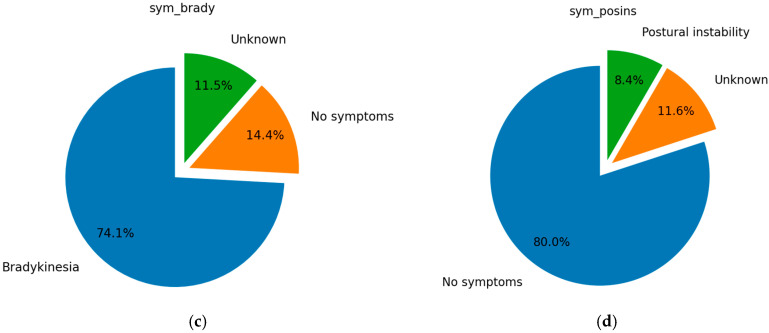
The participants’ symptom profiles and treatment statuses distribution: (**a**) initial symptom (at diagnosis)—resting tremor; (**b**) initial symptom (at diagnosis)—rigidity; (**c**) initial symptom (at diagnosis)—bradykinesia; (**d**) initial symptom (at diagnosis)—postural instability.

The pie charts depicting tremor, rigidity, bradykinesia, and postural instability in Figure 3 illustrate the clinical diversity within the PD cohort used in this study. Although these variables are not considered further as features for ML model training, they provide a comprehensive overview of the participants’ symptom profiles.

The pie charts illustrate the symptomatic distribution of PD within the dataset. Resting tremor, rigidity, and bradykinesia are the most prevalent symptoms, with bradykinesia affecting 83.8% of the subjects, followed by rigidity at 76.2%, and resting tremor at 69.2%. In contrast, postural instability is less common, with only 8.4% affected. This symptomatic breakdown suggests a predominantly PD-diagnosed cohort, emphasizing the need to address class imbalance in diagnostic models. Moreover, the charts highlight the significance of various motor symptoms in PD diagnosis, indicating that the dataset primarily consists of patients with advanced motor symptoms. The presence of advanced motor symptoms in the dataset, such as bradykinesia, rigidity, and resting tremor, is indicative of significant alterations in brain structures associated with PD. These motor symptoms typically arise due to dysfunctions in the basal ganglia circuitry, particularly in the direct and indirect pathways that regulate movement. Imaging techniques like DATSCAN can be particularly useful in such cases. DATSCAN is a SPECT imaging method that visualizes the DAT density in the brain, providing an indirect measure of the integrity of dopaminergic neurons. Given that motor symptoms emerge from substantial neuronal loss and changes in brain structure, DATSCAN imaging can help detect these changes even before severe motor symptoms become apparent. Thus DATSCAN could serve as a robust diagnostic tool, confirming the extent of dopaminergic loss and providing a more accurate diagnosis.

**Figure 4:**
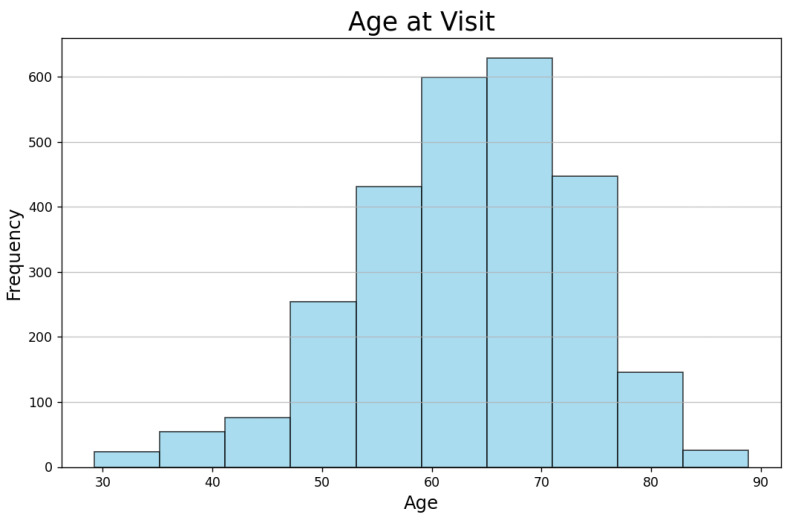
The participants’ age at enrollment in the PPMI project.

**Figure 5:**
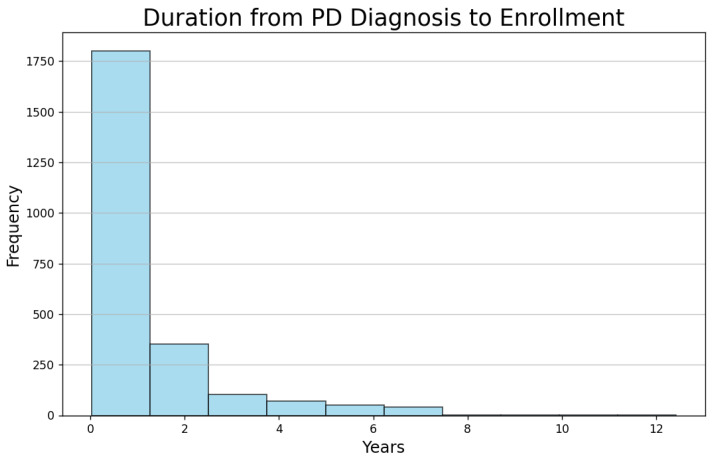
The duration from PD diagnosis to enrollment in the PPMI project.

Figure 4 illustrates the age distribution of participants at the time of their enrollment in the PPMI project, while Figure 5 depicts the time interval between their PD diagnosis and enrollment in the project.

The majority of subjects are in the age range of 60 to 75 years. This suggests that the dataset predominantly includes older individuals, which aligns with the typical age of onset and progression of PD. The peak frequency is around 65–70 years, highlighting that this age group is the most represented in the study. A significant number of subjects were enrolled in the study shortly after their PD diagnosis, with the majority enrolling within the first year. This indicates that the dataset mainly includes newly diagnosed PD patients, which could be valuable for studying PD and its progression.

For PD diagnosis purposes, 14 features are considered from the curated PPMI dataset. These features are taken from DATSCAN images based on information from the study [36]. It was shown above that DAT SPECT imaging is a suitable biomarker for demonstrating PD progression, and it has a high sensitivity in the early stages of PD. Chosen features are shown in Table 1.

In Table 1 above, 13 out of 14 parameters are constructed using a combination of the following DATSCAN parameters: DATSCAN_CAUDATE_L, which is the SBR of the left caudate small brain region of interest referenced to the occipital lobe; DATSCAN_CAUDATE_R, which is the SBR of the right caudate small brain region of interest referenced to the occipital lobe; DATSCAN_PUTAMEN_L, which is the SBR of the left putamen small brain region of interest referenced to the occipital lobe; and DATSCAN_PUTAMEN_R, which is the SBR of the right putamen small brain region of interest referenced to the occipital lobe.

The process of calculation of SBR from a reconstructed DATSCAN is described in [39]. For each scan, putamen region of interest (ROI), striatum ROI, and occipital ROI are determined. SBR is defined as the difference between the count density of caudate or putamen ROIs and the count density of the occipital ROI, divided by the count density of the occipital ROI. Occipital ROI is used to calculate the background count density [40].

### 3.2. Classification Algorithms

Various ML classification algorithms are being considered to identify biomarkers among the 14 selected features extracted from DATSCAN images and develop an effective algorithm for PD detection tasks. Classification is a type of supervised ML technique where the model is trained to predict the accurate label for a given input data. This technique is extensively employed by numerous researchers in the medical field, particularly in PD diagnosis. This research is dedicated to PD diagnosis tasks; thus, binary classification is utilized.

The selection of classifiers for this study was influenced by a comprehensive review detailed in manuscript [23], which analyzed 82 ML studies focused on PD diagnosis, published between 2012 and November 20, 2020. The most frequently utilized classifiers include decision tree classifier, random forest classifier, boosted trees, XGBoost, AdaBoost, k-nearest neighbors classifier (Kneighbors classifier), linear discriminant analysis (LDA), logistic regression, support vector machine (SVM), and naïve Bayes (NB).

Key findings from the review include the following:

- SVM: 72 studies evaluated SVM, reporting accuracies ranging from 63.6% to 99.42%, with the highest reported accuracy in [41];
- AdaBoost: 13 studies achieved accuracies between 54.17% and 98.95%, with [42] obtaining the highest value;
- Logistic regression: 24 studies showed accuracies from 43.9% to 96.55%, with the peak performance by [43];
- Random forest classifier: 32 studies reported accuracies from 64.0% to 99.99%, with [44] achieving the highest;
- XGBoost: investigated in 5 studies, with accuracies ranging from 69.9% to 94.4%, the best being reported by [45];
- Naïve Bayes: 19 studies noted accuracies between 64.0% and 96.88%, with [44] achieving the highest;
- Kneighbors classifier: 25 studies recorded accuracies from 50.0% to 98.5%, with [46] reaching the highest;
- Decision tree classifier: 8 studies reported accuracies between 66.0% and 87.21%, with [47] achieving the highest;
- Boosted trees: 4 studies noted accuracies between 93.88% and 100.0%, with [44] achieving the highest.

Specifically, for SPECT imaging, 5 studies used this modality to diagnose PD [20,48,49,50,51]. One study utilized Tc-Trodat-1 SPECT [48], one used DAT scan SPECT [20], and three used FP-CIT SPECT [49,50,51]. The PPMI database was employed in three studies, with classification performed using RF, boosted trees, LDA, LR, SVM, and NB. Accuracy rates ranged from 82.2% to 97.29%, with the highest achieved using SVM [51]. Sensitivity ranged from 81.8% to 97.3%, with SVM leading, while specificity ranged from 77.5% to 100%, peaking for SVM and LR [48].

Thus, the classifiers chosen for this study were selected based on their proven effectiveness in previous researches.

According to this, to build the best model the following classifiers are considered:

1. SVC;
2. Decision tree classifier;
3. KNeighborsClassifier;
4. Logistic regression classifier;
5. Stochastic gradient descent classifier (SGDClassifier);
6. AdaBoost classifier;
7. Random forest classifier.

### 3.3. Machine Learning Process

To build the best model for PD diagnosis, multiple classifiers are used. The dataset is randomly split into a training set and a test set in a 9:1 ratio. This ratio is recommended for datasets with fewer than 10,000 samples as it optimizes the training set size while retaining an adequately large test set to assess the model’s generalization capabilities effectively. We employ cross-validation and grid search techniques to train the classification models on the training set. GridSearchCV is a systematic method for optimizing model performance by exploring a predefined grid of hyperparameter combinations [52]. In our approach, GridSearchCV is used to optimize hyperparameters and improve model performance. Instead of simply splitting the data into training and validation sets once, we perform multiple splits [53] using repeated stratified k-fold cross-validation [54], which in our case divides the data into 10 folds and repeats this process 10 times with different random splits. This ensures that each model configuration is evaluated across different, randomized data subsets, which provides a more reliable performance estimate. Repeated stratified k-fold cross-validation is crucial when dealing with imbalanced datasets, like ours, which contains more PD cases than healthy controls. The main issue with imbalanced datasets is that certain classes may dominate the model’s learning process, leading to biased predictions. If the folds were randomly split without stratification, the proportion of PD and healthy controls cases could vary significantly between folds, leading to inaccurate model evaluation and training. By preserving the class distribution in each fold through stratification, we ensure that both PD and healthy controls observations are adequately represented in every split, allowing the classifier to learn about both classes evenly. This improves the stability of model training and the reliability of evaluation metrics, such as accuracy, ROC, or F1 score. In this research, grid search usage along with cross-validation technique play a pivotal role in systematically optimizing hyperparameters for each classifier. Grid search involves a comprehensive search through all predefined hyperparameter combinations, while cross-validation ensures that each combination is evaluated across multiple data splits. This approach allows us to reliably estimate model performance and select the most suitable configuration for PD diagnosis. Given the large number of models trained with varying hyperparameters, this technique is essential for identifying the optimal model while preventing overfitting. By using the area under the ROC curve as the primary evaluation metric—a metric particularly suited for binary classification tasks like PD diagnosis—we capture a balanced view of sensitivity and specificity. This ensures that the selected model performs robustly across different subsets of the data, enhancing its generalizability to unseen cases. This method has become a standard in research for selecting high-performing models, as seen in studies such as [1].

Additionally, we assess feature importance using several methods:

- Model-based feature importance—leverages the internal mechanisms of models to rank features based on their contribution to reducing prediction error [55,56];
- Recursive feature elimination with cross-validation (RFECV) [57]—recursively removes the least-important features, retraining the model each time to determine how feature elimination impacts performance. By incorporating cross-validation, RFECV ensures robust selection of the most relevant features across different data splits;
- Permutation importance—evaluates feature importance by measuring the impact on model performance when the values of each feature are randomly shuffled. A significant drop in performance indicates that the feature is essential for accurate predictions [58];
- LIME technique [59].

LIME is a technique used to explain the predictions of complex ML models. It works by approximating the behavior of the model locally around a particular prediction, using a simpler, interpretable model, such as linear regression. This allows us to understand the contribution of individual features to the model’s decision for a specific observation. LIME works by perturbing the features of an observation and observing how these changes affect the model’s predictions.

The main steps of the algorithm are as follows:

- Perturbation: It generates a new dataset of samples by slightly modifying the feature values of the observation being explained. These perturbations help create a local neighborhood around the observation;
- Model prediction: The original model is used to predict outcomes for each of these perturbed samples, allowing LIME to observe how small changes in feature values affect the predictions;
- Weighting: LIME assigns weights to these perturbed samples based on their proximity to the original observation. This ensures that the explanation is more influenced by samples that are closer to the original data point;
- Interpretable model fitting: An interpretable model, such as a linear regression or decision tree, is trained on this weighted dataset. This surrogate model approximates the behavior of the complex model in the local neighborhood of the observation;
- Feature importance extraction: The coefficients of the interpretable model indicate the importance of each feature in the original model’s prediction. These can be used to create explanations that are human-understandable.

In this study, we utilize LIME to explain the predictions of the selected classifier by generating local explanations for every observation in the test set. By analyzing the contributions of individual features to each prediction, we obtain localized insights specific to each sample. After computing these local explanations, the feature importances are averaged across the entire test set, which provides a broader view of how each feature influences the model’s overall decision-making process, giving us a more reliable measure of feature relevance.

By integrating feature importance derived from multiple methods, including model-based feature importance, RFECV, and permutation importance, we generate prioritized lists of features based on each method’s output. These lists are then combined to create an averaged ranking, reflecting the consensus across the different approaches. LIME, which provides a local interpretation of model predictions, is then used to validate this aggregated feature ranking. Through this process, we ensure a more robust and comprehensive identification of the most significant features, ultimately aiding in the selection of the most appropriate biomarker for distinguishing between PD and healthy controls with greater precision.

The decision to use a LIME explainer was mainly influenced by its success in other research, particularly in medical imaging tasks similar to PD diagnosis. For instance, in manuscript [20] involving VGG-16 and CNNs, LIME was used to explain predictions made by DL models. This approach was demonstrated to be effective in medical domains like image classification, where explainability is crucial for building trust in AI-based systems. The use of LIME in these studies highlighted its ability to provide clear and actionable insights, even when dealing with complex models. This made it an ideal choice for the current research, where we aim to improve the interpretability of an AdaBoost model that classifies PD from healthy controls using complex DATSCAN-derived parameters.

**Figure 6:**
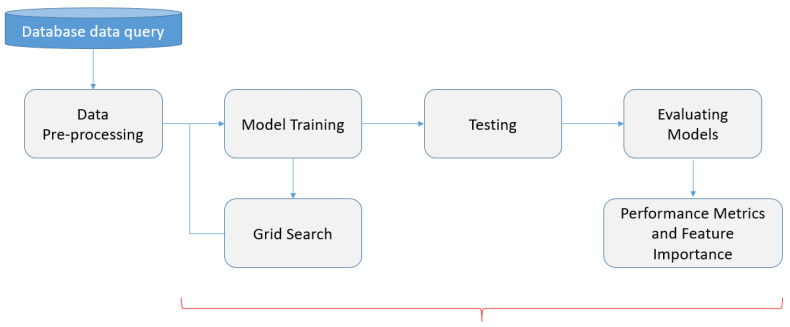
The ML process.

The entire process of building the optimal model and identifying the most relevant biomarker is illustrated in Figure 6.

This ML process can be outlined in the following steps:

- Step 1: database data query;
- Step 2: data preprocessing;
- Step 3: model training, grid search, and choosing hyperparameters;
- Step 4: testing, evaluating models, performance metrics, and feature importance.

To store data in a well-organized manner, the PostgreSQL [60] tool is used. Each table represents a single file provided by PPMI study data. The features for this research are selected from PPMI_Curated_Data_Cut_Public_rev table from curated data cuts.

Then in the data preprocessing step, all measurements containing missing data are excluded from the dataset. Only the measurements corresponding to class 1 (PD participant) and 2 (healthy controls) are chosen as a prerequisite to the binary classification task. Class 1 (PD participant) is then relabeled as 0, and class 2 (healthy controls) is relabeled as 1. The obtained dataset is then split into a training set and a test set in a 9:1 ratio, respectively. After that, for each set a feature scaling is performed using standard scaler [61]. This step enhances algorithm performance, facilitates interpretability, and ensures robustness to outliers. Also, as a preprocessing step, histograms of features and Pearson correlation coefficients are built to understand the internal data structure.

To build the best model in terms of performance metrics and to find the most important feature as a biomarker classifying between PD and healthy controls participants, the model training and choosing hyperparameters procedures are performed using grid search and cross-validation techniques for the SVC, DecisionTreeClassifier, KneighborsClassifier, LogisticRegression, SGDClassifier, AdaBoostClassifier, and RandomForestClassifier algorithms.

For each algorithm, the training procedure consists of the same steps:

- A grid of hyperparameters for a model is defined;
- The number of folds and number of repetitions are defined for repeated stratified k-fold cross-validation. The dataset is split into training and testing sets in a way each fold is stratified (containing the same percentage of samples of each target class);
- For each parameter combination and each fold, the model is trained on the training set and evaluated on the validation set by calculating the performance metric. These metrics are aggregated then across all repetitions and folds for each parameter combination. As a performance metric, ROC score is chosen;
- The parameter combination that maximizes the aggregated performance metric is selected.

For the built models with selected parameters combination, the following metrics are calculated on the test set: TP is true positive, TN is true negative, FP is false positive, FN is false negative, TPR is true positive rate (recall), FPR is false positive rate.

For the built models, coefficient and feature importance scores are taken as feature importance indicators. For all classifiers, the permutation importance method is applied as an additional source of feature importance information. Recursive feature elimination using the cross-validation method is also applied for the KNeighborsClassifier and SGDClassifier to obtain feature importances. Then samples are selected from a test set, and the LIME method is applied to obtain average feature importances. Comparing computed metrics and feature importances, the best model is selected along with the set of features that have the greatest impact on the classification of PD and healthy controls patients.

## 4. Results

### 4.1. Data Preprocessing Step

**Figure 7:**
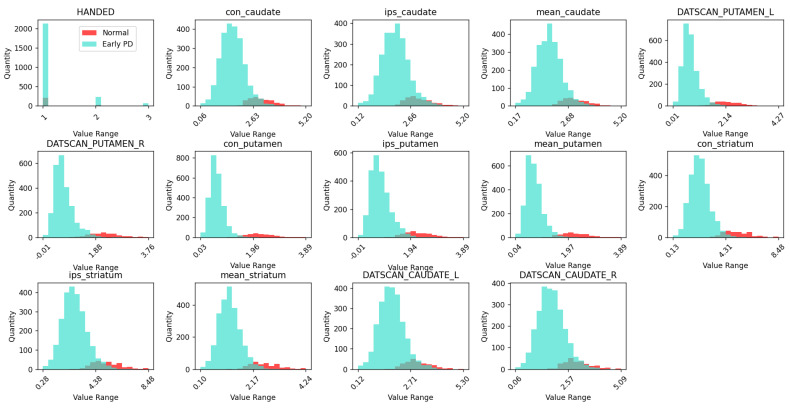
Histograms of features. HANDED 1—right, 2—left, 3—mixed. The rest of the values are unitless SBRs.

Figure 7 shows the histogram of the features that is a result of the preprocessing step of the ML process depicted in Figure 6. It is visible in Figure 7 that there are four features for which distributions between healthy controls patients and patients with PD significantly differ. These four features are con_putamen, mean_putamen, DATSCAN_PUTAMEN_L, and DATSCAN_PUTMEN_R. The PD patients have lower scores in all of the four features. The observed decrease in SBR values for features such as con_putamen, mean_putamen, DATSCAN_PUTAMEN_L, and DATSCAN_PUTAMEN_R in PD patients is consistent with expected outcomes. This decline is indicative of reduced DAT activity in the Put regions, a hallmark of PD. Dopaminergic neuron degeneration, particularly in the SNc, leads to decreased DA levels in the striatum, which includes the Put. Consequently, the SBR values, which reflect the density of functioning DAT, are significantly lower in PD patients compared to healthy controls. These findings reinforce the validity of using these specific features to discriminate between PD patients and healthy individuals, highlighting the critical role of dopaminergic dysfunction in the pathophysiology of PD.

From these histograms, class imbalances are also evident, which are addressed by using stratified k-fold cross-validation. This method ensures that each fold of the dataset maintains the same proportion of class labels, thus providing a more reliable performance evaluation of the ML models. The stratified k-fold cross-validation helps mitigate the impact of class imbalance, leading to more robust and generalizable models for PD detection.

**Figure 8:**
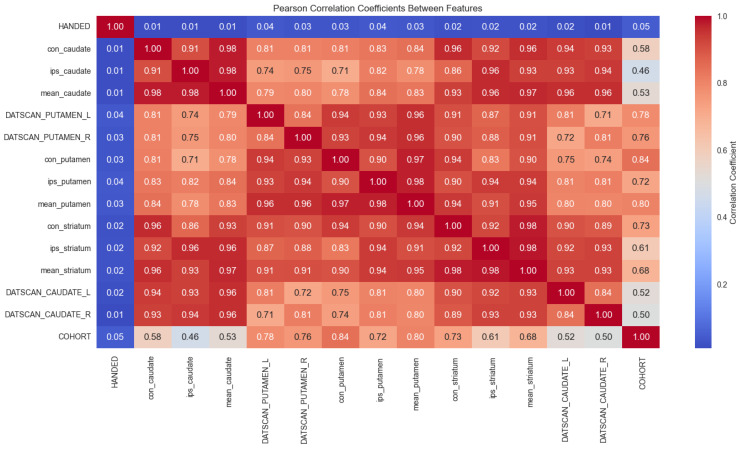
The Pearson correlation coefficients. HANDED 1—right, 2—left, 3—mixed. COHORT 1—healthy controls, 0—PD. The rest of the values are unitless SBRs.

The Pearson correlation coefficient among all the features is shown in Figure 8.

The features DATSCAN_CAUDATE_L, DATSCAN_CAUDATE_R, DATSCAN_PUTAMEN_L, and DATSCAN_PUTMEN_R are correlated with each other. As expected, the highest correlation is observed between con_putamen, ips_putamen, mean_putamen, DATSCAN_PUTAMEN_L, and DATSCAN_PUTMEN_R features. Another highly correlated group of features includes con_caudate, ips_caudate, mean_caudate, DATSCAN_CAUDATE_L, and DATSCAN_CAUDATE_R features. This correlation is indicative of the systemic reduction in DAT availability across these regions in PD, highlighting the widespread impact of the disease on the striatum. During the preprocessing step, we observed that several DATSCAN-related features were highly correlated with each other, indicating potential redundancy. This was further confirmed by the variance inflation factor (VIF) analysis [62], which shows that several features exhibited multicollinearity, meaning they provide overlapping information that can affect model performance and interpretation. In this case, some features might be excluded from the final model, simplifying the model while preserving its predictive power. One of the most significant observations is that con_putamen exhibits the highest correlation with the COHORT value, suggesting it is potentially the most informative feature. This high correlation aligns with the biological relevance of the putamen region in PD, as it is heavily impacted by dopamine depletion. Given the high correlation of con_putamen with the COHORT, it can be selected as the primary biomarker for model training. Other features, particularly those with high inter-correlations, may be excluded to avoid multicollinearity. This approach ensures that the model remains both interpretable and robust, focusing on the most biologically relevant feature for PD diagnosis while maintaining predictive accuracy and reducing redundant information.

### 4.2. ML Algorithms Training Step

Table 2 presents the top-performing classifiers identified through the grid search technique, including their ROC scores and hyperparameters. It shows that, on the training set, the logistic regression and SVC classifiers achieved the highest ROC scores.

All the metrics computed on test data for each selected classifier are presented in Table 3. The SVC classifier demonstrates the highest ROC score (99.98%), the highest accuracy (99.25%), precision score (96.15%), and F1 score (96.15%). This suggests that SVC is highly capable of distinguishing between healthy controls (class 1) and Parkinson’s disease (class 0) with minimal misclassification. The decision tree classifier has perfect recall (100.00%) but lower precision (86.67%), indicating that while it captures all positive cases, it also generates a relatively high number of false positives. Logistic regression and random forest classifier both show a balanced performance across all metrics, particularly in terms of F1 score and G-Mean, indicating that they handle the trade-off between precision and recall well. SGD classifier has the lowest performance across most metrics, particularly in G-Mean and F1 score, indicating it may struggle with correctly classifying both classes. Looking into the SVC model in more detail, it can be seen that SVC with regularization parameter set to 100, the degree of the polynomial kernel function set to two, and the type of kernel used by the SVC set to polynomial have the highest mean ROC metric values computed on folds during the cross-validation procedure. This makes this model the most accurate in the PD detection task.

**Figure 9:**
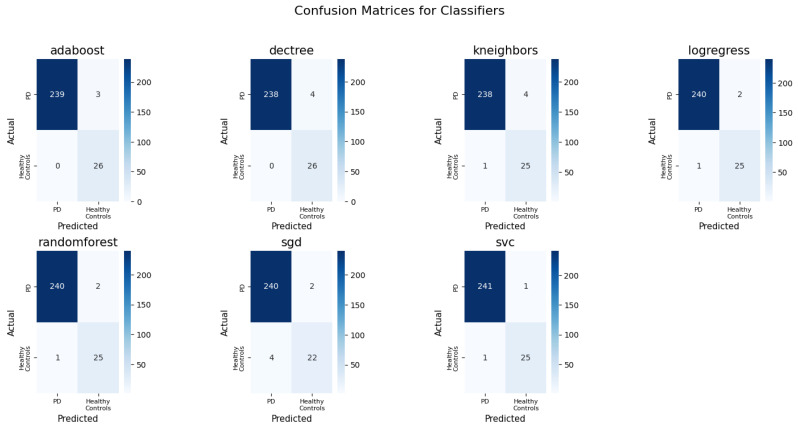
Confusion matrices for classifiers trained on all features and computed on a test set.

The confusion matrices presented in Figure 9 provide insights into how well each classifier performed on the test set. Since these results are computed on the test set, it validates that the models are not overfitting and are generalizing well to unseen data. This gives confidence that the models are robust for future unseen patients. Most of the classifiers perform quite well in distinguishing PD from healthy controls. The true positive rate for healthy controls is relatively high across models, which is crucial in medical diagnostics. False negatives (patients with the disease wrongly classified as non-disease) are critical in medical scenarios, and, fortunately, all models show very few false negatives. The comparison of confusion matrices is helpful in selecting the most accurate and reliable model for the final deployment. Based on the matrices, logistic regression, SVC, and random forest seem to have the most balanced performance with the least misclassifications.

### 4.3. Feature Importance Identification Step

**Figure 10:**
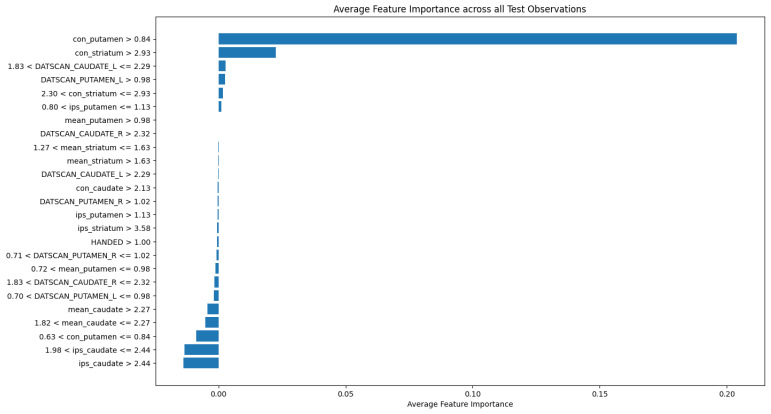
Feature importances using the LIME method.

Through the feature importances identification step in our ML process, we obtained feature importances. Figure 10 displays an average feature importance chart generated by the LIME method using all observations from a test set for the AdaBoost classifier. Each bar in the chart represents the magnitude of a feature’s impact under specific conditions, with the direction (positive or negative) indicating whether the feature increases or decreases the likelihood of the model predicting a specific class.

According to Figure 10, con_putamen shows the highest impact on predicting target values, suggesting it could serve as a biomarker distinguishing between PD and healthy controls patients.

**Figure 11:**
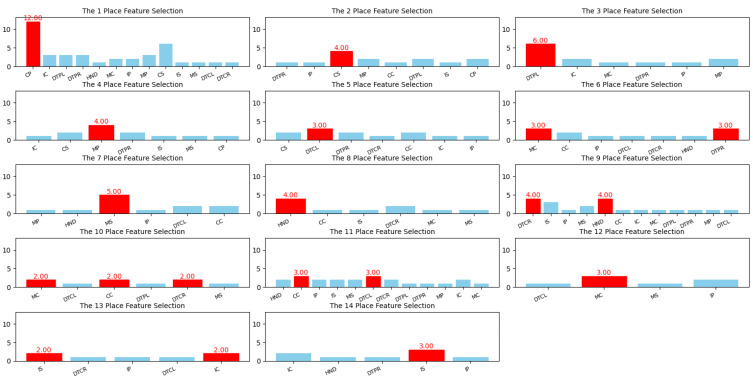
The distribution of feature importances.

Figure 11 shows the distribution of obtained feature importances using various methods. From these charts, the most important feature ranking was obtained, which is shown in Table 4.

Based on Figure 11 and Table 4, con_putamen can be used as a discriminating feature between PD and healthy controls patients.

Given the results presented, con_putamen can be identified as the primary biomarker, while other features should be excluded to mitigate multicollinearity. Considering only the con_putamen parameter alone for training the same classification algorithms, Table 5 presents the top-performing classifiers identified through the grid search technique, including their ROC scores and hyperparameters. It shows that, on the training set, the logistic regression, SGD, and SVC classifiers achieved the highest ROC scores.

Based on Table 5, for most classifiers, the ROC score shows only a slight decrease (if any) when moving from 14 features to just the con_putamen feature. This indicates that con_putamen is a particularly strong predictor and plays a crucial role in the model’s performance. The decision tree classifier uniquely performs better with the single con_putamen feature than with all 14 features, suggesting that in this case, simpler models might be more effective when using highly informative features. SVC, logistic regression, SGD classifier, and AdaBoost all demonstrate robustness and consistency in their performance regardless of the number of features, showing that these models are less sensitive to the inclusion of additional features. Overall, this comparison underscores the significance of the con_putamen feature and suggests that while additional features can offer incremental improvements, they are not always necessary for strong model performance, depending on the model used.

Considering only the con_putamen parameter for training the same classification algorithms, the classification metrics on the test set are shown in Table 6.

Based on Table 6, SVC, logistic regression, and random forest show better performance with 14 features, indicating that these models benefit from a richer feature set to achieve balanced classification. Decision tree and SGD classifier show improved precision with con_putamen but at the cost of lower recall and F1 scores, indicating that while they are more precise, they might miss some positive cases. Kneighbors and AdaBoost classifiers perform similarly or slightly better with just con_putamen, highlighting the strength of this feature for these models. Overall, while most models benefit from the inclusion of additional features, con_putamen proves to be a strong single feature that can lead to competitive performance on its own. While a comprehensive set of features can provide marginal benefits, con_putamen proves to be particularly critical, as evidenced by the minimal impact on most classifiers’ performance when other features are excluded. This reinforces the importance of identifying and utilizing key features in medical diagnosis and predictive modeling to achieve optimal results with potentially fewer variables. The AdaBoost classifier demonstrated the highest accuracy and ROC score when trained solely on the con_putamen feature.

**Figure 12:**
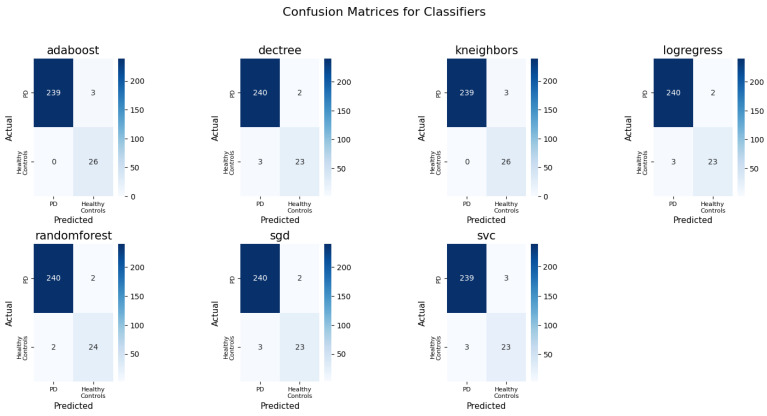
Confusion matrices for classifiers trained on con_putamen feature and computed on test set.

The confusion matrices for the algorithms trained on a con_putamen feature and computed on a test set are shown in Figure 12. Most of the classifiers perform quite well in distinguishing PD from healthy controls. The true positive rate for healthy controls is relatively high across models, while there are very few false negatives. Based on the matrices, AdaBoost seems to have the most balanced performance with the least misclassifications.

To assess and validate the performance of the ML models on the selected dataset, we calculated key evaluation metrics, such as accuracy, precision, recall, F1 score, and ROC AUC, along with their respective confidence intervals. Confidence intervals offer a statistical range within which the true value of a metric is likely to fall, accounting for variability in the dataset. These intervals are essential for evaluating the robustness and reliability of the models, particularly in critical applications like medical diagnostics. We utilized bootstrapping [63] to determine the confidence intervals for each metric, which is shown in Table 7. This technique involves repeatedly resampling the test dataset with a replacement, computing the metrics for each sample, and extracting the percentiles. By incorporating variability across resampled datasets, this method provides a comprehensive view of the stability of model performance under different sample distributions. The results reveal that all models demonstrated consistently high performance, with accuracy, recall, and ROC AUC exceeding 95%. Notable variations in precision and F1 score among models reflect their differing capacities to address imbalances or specific misclassification scenarios. The narrow confidence intervals further emphasize the reliability and robustness of these metrics. Overall, the findings confirm that the models are highly effective in classifying PD symptoms using the con_putamen feature, with AdaBoost and k-nearest neighbors models achieving slightly higher recall and F1 scores. These results underscore the potential applicability of these models in real-world diagnostic scenarios.

**Figure 13:**
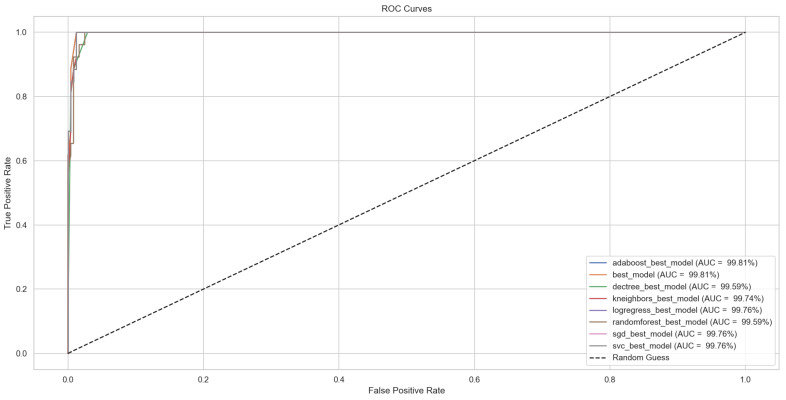
ROC curves for the models along with AUC scores.

The ROC curve shown in Figure 13 was created to evaluate and compare the ability of different ML models to distinguish between PD symptoms and non-symptomatic cases. This is particularly important in medical diagnostics, where minimizing false negatives is critical. The ROC curve plots the true positive rate against the false positive rate at various classification thresholds, allowing a detailed assessment of model sensitivity and specificity. The area under the curve (AUC) serves as a single numerical metric to summarize model performance, with values closer to 1 indicating better discrimination ability. In this case, all tested models achieved exceptionally high AUC values, exceeding 99%, demonstrating their strong ability to separate the two classes. Notably, the AdaBoost model slightly outperformed the others with an AUC of 99.81%. The tightly clustered ROC curves reflect the models’ consistency and reliability across different thresholds. These results confirm the suitability of the selected models for diagnostic applications, ensuring accurate detection of PD symptoms while minimizing the risk of misclassification.

**Figure 14:**
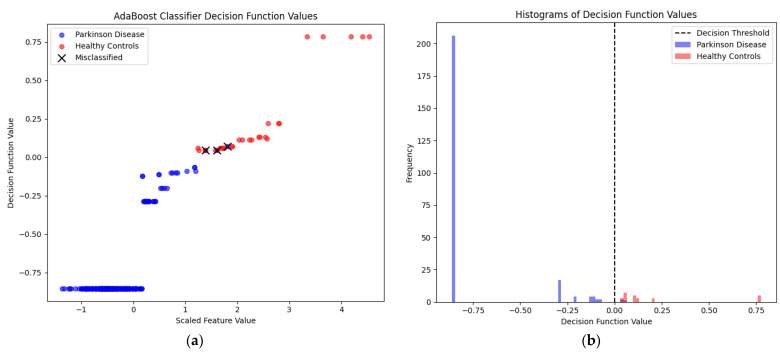
AdaBoost classifier decision function values: (**a**) decision function values for PD and healthy controls observations for single con_putamen feature; (**b**) histograms of decision function values for PD and healthy controls observations for single con_putamen feature.

Figure 14 presents the AdaBoost classifier decision function values for observations from a test set containing only a single con_putamen feature.

The scatter plot visualizes the decision function values of a trained AdaBoost classifier against the scaled values of a con_putamen feature. The X-axis represents the scaled values of this feature, while the Y-axis displays the decision function values, which indicate how confidently the model classifies each sample. Only three out of two hundred sixty-eight observations in the test set, originally labeled as healthy controls, were incorrectly classified as PD.

The histogram plot shows the distribution of decision function values for PD and the healthy controls class. The X-axis represents the decision function values, and the Y-axis represents the frequency of these values. The vertical dashed line indicates the decision threshold used by the classifier. This threshold separates the decision function values into the two classes.

Finally, we separated the initial dataset by gender, examining both male and female patients to perform the entire ML process for each group. The analysis aimed to explore potential differences in classification performance between male and female participants and to explore any gender-specific differences between features used for PD identification. Performance measures for all features and the con_putamen feature in male and female patients is shown in Table 8. The results indicate that con_putamen remains a significant biomarker for both genders. Notably, for females, the SVC emerges as the top performer based on the ROC score, while for males, the Kneighbors classifier achieves the highest ROC score. Ultimately, the classifiers and the con_putamen biomarker can be effectively utilized for both males and females with comparable quality.

## 5. Discussion

As the primary changes in PD patients occur in brain structures, with other symptoms being secondary, we chose to focus on brain structures and consequently on DATSCAN-related parameters in this research. This study emphasizes the con_putamen feature’s potential as a useful biomarker for PD diagnosis. The use of ML techniques, particularly ensemble methods like AdaBoost, has demonstrated high accuracy and reliability of PD diagnosis. By focusing on the con_putamen feature, which has shown strong discriminative power between healthy individuals and those with PD, the presented AdaBoost model achieved a high level of accuracy and ROC score.

The primary limitation of this study is that DATSCAN imaging, while useful for confirming a diagnosis, may not be suitable for detecting PD in its earliest stages. By the time changes in DAT availability are visible on a DATSCAN, around 50% or more of dopamine-producing neurons have already degenerated, meaning significant damage has occurred before imaging can identify the disease. Thus, although DATSCAN helps differentiate PD from other conditions, it does not serve as an early diagnostic tool. Despite its limitations for very early detection, DATSCAN remains valuable for diagnosing PD in its early phases—before severe motor symptoms become apparent—by detecting reduced DAT availability in the striatum. However, because other neurodegenerative diseases such as multiple system atrophy (MSA) or progressive supranuclear palsy (PSP) also cause DAT loss in the putamen, it can potentially lead to false positives, reducing the specificity of the diagnosis. If a doctor were to use the AdaBoost classifier based solely on the con_putamen SBR from DATSCAN data, the result may not always be fully reliable due to several factors. First, the classifier’s accuracy depends on the quality of the input data and the representativeness of the training data. If the doctor’s dataset differs significantly from the training data (in terms of patient demographics, comorbidities, or image quality), the outcomes may not be as reliable. Additionally, con_putamen is a strong predictor but should not be the sole criterion for diagnosing PD, as other diseases like multiple system atrophy (MSA) or progressive supranuclear palsy (PSP) can also exhibit DAT loss in the putamen. This could reduce the specificity of the model. Therefore, although the AdaBoost classifier is a useful tool for diagnosing PD, it should be complemented by clinical judgment and additional diagnostic tests, particularly for early-stage detection, to avoid over-reliance on a single imaging biomarker.

## 6. Conclusions

PD diagnosis is a complex task that implies the utilization of the most recent medical studies as well as multiple algorithms and biomarkers usage. It is demonstrated that the AdaBoost classifier produces superior results. Based on our analysis, the contralateral putamen emerges as the most significant feature for diagnosing PD. Our research shows that the hemisphere opposite to the symptomatic side of the body consistently exhibits substantial loss of dopaminergic neurons in the putamen. This correlation highlights the critical role of the contralateral putamen in the pathophysiology of PD and underscores its importance as a diagnostic marker. By focusing on this specific brain structure, clinicians can enhance the accuracy of PD diagnosis and potentially improve patient outcomes through targeted interventions.

The main limitation of this study lies in the use of DATSCAN imaging, which, although valuable for confirming a diagnosis, is less effective in detecting PD in its earliest stages—before significant neuronal damage occurs. Furthermore, the classifier’s performance may fluctuate based on the quality and representativeness of the input data.

In future work, we plan to investigate additional classification methods and biomarkers to enhance the proposed model, with the goal of improving the effectiveness of early PD diagnosis. Additionally, we aim to develop a personalized patient care model by leveraging longitudinal data to facilitate early-stage disease detection.

## Figures and Tables

**Table 1:** Features are chosen from the PPMI dataset.

Feature	Description
con_caudate	Contralateral caudate. If a participant is a healthy control, the mean of the left and right caudate values is taken. If a participant is a PD: If DOM SIDE is right, DATSCAN_CAUDATE_L is taken. If DOM SIDE is left, DATSCAN_CAUDATE_R is taken. If DOM SIDE is symmetric, the mean of the left and right caudate values is taken, where DOM SIDE is the side predominantly affected at the onset.
con_putamen	Contralateral putamen. If a participant is a healthy control, the mean of the left and right putamen values is taken. If a Participant is a PD: If DOM SIDE is right—DATSCAN_PUTAMEN_L is taken. If DOM SIDE is left, DATSCAN_PUTAMEN_R is taken. If DOM SIDE is symmetric, the mean of the left and right putamen values is taken, where DOM SIDE is the side predominantly affected at the onset.
con_striatum	Contralateral striatum. If a participant is a healthy control, the mean of the four DATSCAN variables is taken. If a participant is a PD: If DOM SIDE is right, DATSCAN_PUTAMEN_L + DATSCAN_CAUDATE_L is taken. If DOM SIDE is left, DATSCAN_PUTAMEN_R + DATSCAN_CAUDATE_R is taken. If DOM SIDE is symmetric, the mean of the four DATSCAN variables is taken, where DOM SIDE is the side predominantly affected at the onset.
DATSCAN_CAUDATE_L	Left caudate, striatal binding ratio of the left caudate small brain region of interest referenced to the occipital lobe.
DATSCAN_CAUDATE_R	Right caudate, striatal binding ratio of the right caudate small brain region of interest referenced to the occipital lobe.
DATSCAN_PUTAMEN_L	Left putamen, striatal binding ratio of the left putamen small brain region of interest referenced to the occipital lobe.
DATSCAN_PUTAMEN_R	Right putamen, striatal binding ratio of the right putamen small brain region of interest referenced to the occipital lobe.
ips_caudate	Ipsilateral caudate. If the participant is a healthy control, the mean of the left and right caudate values is taken. If a participant is a PD: If DOM SIDE is right, DATSCAN_CAUDATE_R is taken. If DOM SIDE is left, DATSCAN_CAUDATE_L is taken. If DOM SIDE is symmetric, the mean of the left and right caudate values is taken.
ips_putamen	Ipsilateral putamen. If a participant is a healthy control, the mean of the left and right putamen values is taken. If a participant is a PD: If DOM SIDE is right, DATSCAN_PUTAMEN_R is taken. If DOM SIDE is left, DATSCAN_PUTAMEN_L is taken. If DOM SIDE is symmetric, the mean of the left and right putamen values is taken.
ips_striatum	Ipsilateral striatum. If a participant is a healthy control, the mean of the four DATSCAN variables is taken. If a participant is a PD: If DOM SIDE is right, DATSCAN_PUTAMEN_R + DATSCAN_CAUDATE_R is taken. If DOM SIDE is left, DATSCAN_PUTAMEN_L + DATSCAN_CAUDATE_L is taken. If DOM SIDE is symmetric, the mean of the four DATSCAN variables is taken.
mean_caudate	Mean caudate measure. (DATSCAN_CAUDATE_R + DATSCAN_CAUDATE_L)/2
mean_putamen	Mean putamen measure. (DATSCAN_PUTAMEN_R + DATSCAN_PUTAMEN_L)/2
mean_striatum	Mean striatum measure. (DATSCAN_CAUDATE_R + DATSCAN_CAUDATE_L + DATSCAN_PUTAMEN_R + DATSCAN_PUTAMEN_L)/4
HANDED	Handedness

**Table 2:** Top-performing classifiers identified through the grid search technique.

Model Name	Hyperparameters	Mean (std) ROC Score (%)
SVC	C: 100, degree: 2, kernel: poly	99.79 (0.24)
Decision Tree Classifier	criterion: entropy, max_depth: 4, max_features: sqrt, splitter: best	98.46 (1.45)
Kneighbors Classifier	algorithm: brute, n_neighbors: 15, weights: distance	99.25 (1.13)
Logistic Regression	penalty: l2, solver: saga	99.74 (0.2)
SGD Classifier	learning_rate: optimal, loss: hinge, max_iter: 500	99.70 (0.22)
AdaBoost Classifier	algorithm: SAMME, n_estimators: 50, learning_rate: 0.5	99.35 (0.88)
R Random Forest Classifier	criterion: entropy, n_estimators: 200	99.41 (0.91)

**Table 3:** Performance measures on the test set for the selected ML models.

Model Name	Accuracy Score (%)	Precision Score (%)	Recall Score (%)	F1 Score (%)	Geometric Mean Score (%)	ROC Score (%)
SVC	99.25	96.15	96.15	96.15	97.86	99.98
Decision Tree Classifier	98.51	86.67	100.00	92.86	99.17	99.36
Kneighbors Classifier	98.13	86.21	96.15	91.00	97.24	99.67
Logistic Regression	98.88	92.59	96.15	94.34	97.65	99.86
SGD Classifier	97.76	91.67	84.62	88.00	91.61	99.86
AdaBoost Classifier	98.88	89.66	100.00	94.55	99.38	99.70
Random Forest Classifier	98.88	92.59	96.15	94.34	97.65	99.90

**Table 4:** The ranking of features according to their importance.

Priority	Feature	Priority	Feature
1	con_putamen	8	ips_striatum
2	con_striatum	9	con_caudate
3	DATSCAN_PUTAMEN_L	10	DATSCAN_CAUDATE_L
4	mean_putamen	11	mean_striatum
5	ips_caudate	12	mean_caudate
6	DATSCAN_PUTAMEN_R	13	DATSCAN_CAUDATE_R
7	ips_putamen	14	HANDED

**Table 5:** Top-performing classifiers for con_putamen feature identified through the grid search technique.

Model Name	Hyperparameters	Mean (std) ROC Score (%)
SVC	C: 0.1, degree: 2, kernel: linear	99.67 (0.28)
Decision Tree Classifier	criterion: Gini, max_depth: 4, max_features: sqrt, splitter: random	99.38 (0.73)
Kneighbors Classifier	algorithm: brute, n_neighbors: 20, weights: uniform	99.23 (0.96)
Logistic Regression	penalty: l1, solver: liblinear	99.67 (0.28)
SGD Classifier	learning_rate: optimal, loss: log_loss, max_iter: 1500	99.67 (0.28)
AdaBoost Classifier	algorithm: SAMME.R, learning_rate: 0.5, n_estimators: 50	99.35 (0.88)
Random Forest Classifier	criterion: Gini, n_estimators: 200	98.26 (1.47)

**Table 6:** Performance measures for con_putamen feature on the test set for the selected ML model.

Model Name	Accuracy Score (%)	Precision Score (%)	Recall Score (%)	F1 Score (%)	Geometric Mean Score (%)	ROC Score (%)
SVC	97.76	88.46	88.46	88.46	93.47	99.76
Decision Tree Classifier	98.13	92.00	88.46	90.20	93.66	99.59
Kneighbors Classifier	98.88	89.66	100.00	94.55	99.38	99.74
Logistic Regression	98.13	92.00	88.46	90.20	93.66	99.76
SGD Classifier	98.13	92.00	88.46	90.20	93.66	99.76
AdaBoost Classifier	98.88	89.66	100.00	94.55	99.38	99.81
Random Forest Classifier	98.51	92.31	92.31	92.31	95.68	99.59

**Table 7:** Performance measures along with their confidence intervals for con_putamen feature on the test set for the selected ML model.

Model Name	Accuracy Score (%) Confidence Interval	Precision Score (%) Confidence Interval	Recall Score (%) Confidence Interval	F1 Score (%) Confidence Interval	ROC Score (%) Confidence Interval
AdaBoost Classifier	98.88 (97.76, 100.0)	89.66 (77.27, 100.0)	100.0 (100.0, 100.0)	94.55 (87.09, 100.0)	99.81 (99.43, 100.0)
Decision Tree Classifier	98.13 (96.23, 99.63)	92.00 (81.25, 100.0)	88.46 (75.0, 100.0)	90.20 (80.00, 97.78)	99.59 (99.05, 99.96)
Kneighbors Classifier	98.88 (97.39, 100.0)	89.66 (77.14, 100.0)	100.00 (100.0, 100.0)	94.55 (86.95, 100.0)	99.74 (99.31, 100.0)
Logistic Regression	98.13 (96.27, 99.63)	92.00 (78.94, 100.0)	88.46 (74.07, 1.0)	90.20 (80.00, 98.11)	99.76 (99.32, 100.0)
Random Forest Classifier	98.51 (96.64, 99.63)	92.31 (80.0, 100.0)	92.31 (80.0 100.0)	92.31 (82.60, 98.51)	99.59 (99.05, 100.0)
SGDClassifier	98.13 (96.27, 99.63)	92.00 (79.31, 100.0)	88.46 (75.99 100.0)	90.20 (80.00, 97.44)	99.76 (99.31, 100.0)
SVC	97.76 (95.89, 99.25)	97.76 (75.74, 100.0)	88.46 (74.98 100.0)	88.46 (78.04, 96.43)	99.76 (99.35, 100.0)

**Table 8:** Performance measures for all features and con_putamen feature in male and female patients.

Case	Model	Hyperparameters	Accuracy (%)	Precision (%)	Recall (%)	F1 Score (%)	ROC (%)
Female all features	SVC	{‘C’: 1, ‘decision_function_shape’: ‘ovr’, ‘degree’: 3, ‘gamma’: ‘scale’, ‘kernel’: ‘linear’, ‘probability’: True}	99.02	100.00	90.00	94.74	99.78
Female con_putamen	SVC	{‘C’: 0.1, ‘decision_function_shape’: ‘ovr’, ‘degree’: 3, ‘gamma’: ‘scale’, ‘kernel’: ‘linear’, ‘probability’: True}	97.06	88.89	80.00	84.21	99.73
Male all features	Random Forest Classifier	{‘bootstrap’: True, ‘ccp_alpha’: 0.0, ‘criterion’: ‘entropy’, ‘max_features’: ‘sqrt’, ‘min_impurity_decrease’: 0.0, ‘min_samples_leaf’: 1, ‘min_samples_split’: 2, ‘min_weight_fraction_leaf’: 0.0, ‘n_estimators’: 50}	98.19	88.24	93.75	90.91	99.60
Male con_putamen	Kneighbors Classifier	{‘algorithm’: ‘auto’, ‘leaf_size’: 30, ‘metric’: ‘minkowski’, ‘n_neighbors’: 10, ‘p’: 2, ‘weights’: ‘uniform’}	98.20	88.24	93.75	90.91	99.67

## Data Availability

Data used in the preparation of this article were obtained from the Parkinson’s Progression Markers Initiative (PPMI) database [(https://www.ppmi-info.org/access-dataspecimens/download-data, accessed on 4 December 2024, RRID:SCR 006431]. For up-to-date information on the study, visit www.ppmi-info.org. This analysis used data openly available from PPMI, obtained from PPMI upon request. Further inquiries can be directed to the corresponding author.
